# Identification of rare germline copy number variations over-represented in five human cancer types

**DOI:** 10.1186/s12943-015-0292-6

**Published:** 2015-02-03

**Authors:** Richard W Park, Tae-Min Kim, Simon Kasif, Peter J Park

**Affiliations:** Bioinformatics Program, Boston University, Boston, MA USA; Center for Biomedical Informatics, Harvard Medical School, 10 Shattuck St, Boston, MA 02115 USA; Department of Medical Informatics, College of Medicine, The Catholic University of Korea, Seoul, 137-701 South Korea; Department of Biomedical Engineering, Boston University, Boston, MA USA; Children’s Hospital Informatics Program, Harvard-MIT Division of Health Sciences and Technology, Cambridge, MA USA

**Keywords:** Array CGH, DNA copy number, CNV association study, Cancer susceptibility

## Abstract

**Background:**

Copy number variations (CNVs) are increasingly recognized as significant disease susceptibility markers in many complex disorders including cancer. The availability of a large number of chromosomal copy number profiles in both malignant and normal tissues in cancer patients presents an opportunity to characterize not only somatic alterations but also germline CNVs, which may confer increased risk for cancer.

**Results:**

We explored the germline CNVs in five cancer cohorts from the Cancer Genome Atlas (TCGA) consisting of 351 brain, 336 breast, 342 colorectal, 370 renal, and 314 ovarian cancers, genotyped on Affymetrix SNP6.0 arrays. Comparing these to ~3000 normal controls from another study, our case–control association study revealed 39 genomic loci (9 brain, 3 breast, 4 colorectal, 11 renal, and 12 ovarian cancers) as potential candidates of tumor susceptibility loci. Many of these loci are new and in some cases are associated with a substantial increase in disease risk. The majority of the observed loci do not overlap with coding sequences; however, several observed genomic loci overlap with known cancer genes including *RET* in brain cancers, *ERBB2* in renal cell carcinomas, and *DCC* in ovarian cancers, all of which have not been previously associated with germline changes in cancer.

**Conclusions:**

This large-scale genome-wide association study for CNVs across multiple cancer types identified several novel rare germline CNVs as cancer predisposing genomic loci. These loci can potentially serve as clinically useful markers conferring increased cancer risk.

**Electronic supplementary material:**

The online version of this article (doi:10.1186/s12943-015-0292-6) contains supplementary material, which is available to authorized users.

## Introduction

The major sources of variation in the genomes of individuals include single nucleotide polymorphisms (SNPs), small insertion or deletions (indels), and larger-scale variations. The large-scale variants may be copy number differences (gains or losses of chromosomal segments) or copy number-neutral changes (such as inversions or balanced chromosomal translocations). Copy number variation (CNV) generally refers to large-scale (>1 kb) chromosomal copy number changes, e.g., amplifications or deletions compared to a reference genome [1], although the size distinction is an artificial one defined by the limitations of previous CNV detection methods. Genome-wide CNV screening methods using high-resolution oligonucleotide-based microarrays and more recently, high-throughput sequencing have accelerated the cataloguing and characterization of large genomic variants.

Initial CNV studies reported a greater than expected variability in genomic CNVs in the normal human population, i.e., a significant fraction of individual human genomes may be different from each other [2-4]. In 2006, the first large-scale population map of CNVs was constructed, with estimates that up to 12% of the human genome may harbor CNVs [3]. Recent updates from the Database of Genomic Variants (DGV) estimate CNVs to encompass up to 22% of the human reference genome, making them the most prevalent type (by size) of genomic variability between individuals [4]. In the early days, the focus of genome-wide association studies (GWAS) was to identify disease-associated SNPs. However, as the array platforms and the algorithms for inferring CNVs from the same arrays have improved, more recent studies have identified a number of germline CNVs as potential susceptibility loci for a range of diseases including infectious, autoimmune, and neuropsychiatric diseases, as well as cancer [5-8].

Multiple germline CNVs have been reported as factors predisposing individuals towards cancer pathogenesis. For example, CNVs at 3p25 and 2p24.3 were associated with the aggressiveness of prostate cancer [9,10]. Deletions and rearrangements in the *BRCA* family of genes have been implicated in breast and ovarian cancers [11,12]. Deletions of *GSTM1* and *GSTT1* were shown to decrease the 5-year cancer survival rates for bladder and prostate cancers in the Dutch general population [13]. A recent large-scale CNV association study revealed that CNVs at 1q21.1 involving the *NBPF* family of genes were found to predispose individuals to neuroblastoma [14].

In this study, we identified recurrent germline CNVs in cancer patients from the Cancer Genome Atlas (TCGA) [15-18] that may be associated with increased susceptibility for cancer. For five major types of human cancers (breast invasive carcinomas [15], colorectal cancers [16], glioblastoma multiforme [17], ovarian serous cystadenocarcinomas [18], and renal cell carcinomas [19]), germline CNV calls from each cancer cohort was compared to a normal control population obtained from an unrelated large GWAS study [20]. Our results provide an initial catalog of germline CNVs that are associated with an individual’s predisposition to specific cancers and may serve as biomarkers in cancer screening.

## Results and discussion

### Study design

The Cancer Genome Atlas (TCGA) has aimed to identify and catalog major cancer-causing genomic changes by profiling 500 patients for each of ~20 cancer types. For each patient, DNA from tumor and matched control were profiled, with peripheral blood as the control in most cases and a non-tumor tissue in a small subset of cases. With the project near completion, it has provided access to an unprecedented amount of genomic profiling data from cancer patients, including exome sequencing for most cases, whole-genome sequencing (~10% of cases in many tumor types), RNA and microRNA expression, DNA methylation at CpG islands, and DNA copy number. To characterize CNVs, every sample was profiled on Affymetrix SNP 6.0 arrays. In the pilot phase of the project, the same samples were also profiled on Illumina and Agilent arrays; later on, low-pass whole-genome sequencing (6-8X) was also utilized but only for a subset of the cases. For this study, we focused on germline copy number profiles estimated from the Affymetrix SNP 6.0 platform because it allows us to examine the largest number of cases as well as having the highest probe density (~1 million probes primarily for SNP detection and another ~1 million for CNVs). We chose cancer types with at least 400 normal samples at the beginning of our study, resulting in a total of 1,779 cases across the five cancer types mentioned earlier. The raw data were downloaded from the Cancer Genome Atlas data portal (https://tcga-data.nci.nih.gov/tcga/).

One of the challenges in our analysis was to identify a proper control dataset. First, it was important to find a dataset with sufficiently large sample size to detect rare variants. Studies from the 1000 Genomes project have found that rare genomic variants vastly outnumber common variants [8], identifying approximately 20,000 CNVs with frequencies down to 1% [21,22]. Without a large enough control set, CNVs identified from TCGA germline samples may include rare variants in the population not related to cancer. Second, assessment of CNVs can be confounded by differences in array platforms and methods of analysis [23]. Thus it was necessary that the control subjects were profiled on the same Affymetrix SNP6.0 platform, processed using the same analytical parameters. Third, analysis results can also be confounded by ethnic backgrounds [23-26]. To minimize this effect, we limited our analysis to individuals of Western European descent, due to low numbers of samples available for other ethnicities, and had to use a control set from a similar population; we also ensured that the results were not spurious due to ethnicity differences using principal component analysis. Finally, we have found that most GWAS studies make genotype calls available but not raw data, even after publication. For the current study, it was necessary to access the raw data, so that we could process both cancer and control data uniformly from the start. After an extensive search for healthy human controls, we converged on the data available from the Myocardial Infarction Genetics Consortium (MIGEN), which had over 3,074 healthy controls generated on the Affymetrix SNP 6.0 platform. The data was obtained from dbGAP and from the investigators on the project. For this dataset, the samples were drawn from six collection sites: Boston, MA; Seattle, WA; Helsinki, Finland; Malmö, Sweden; Barcelona, Spain; and Milan, Italy [20].

### Identification of copy number variable regions

**Figure 1:**
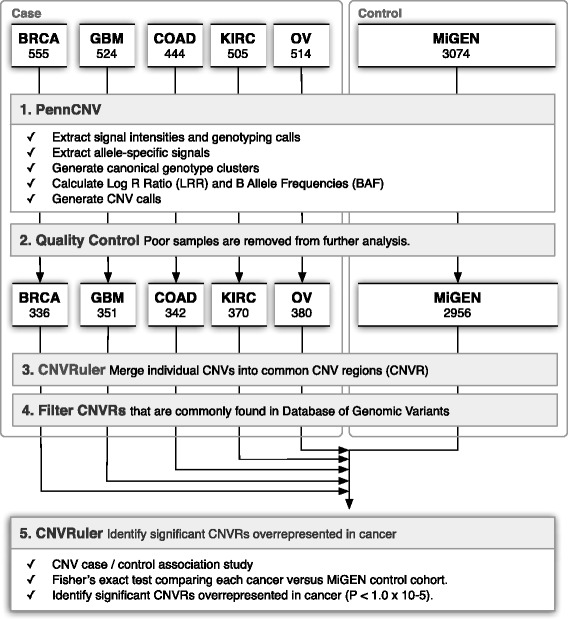
**Workflow for our CNV analysis.** The numbers of samples at various steps are indicated.

An overview of the data and analysis steps are shown in Figure 1. To identify CNVs, we used the PennCNV [27] software package. This algorithm employs a hidden Markov model to segment the total signal intensity for both alleles (log R ratio, or LRR) and allelic intensity ratio between the two alleles (B allele frequency, or BAF) for each probe across the genome. Additional sources of information such as probe spacing and population allele frequency are also incorporated.

Across all five cancers, we identified 88,910 CNVs from 1,779 patients with a median CNV count of 15 gains and 32 losses per patient. The median length of these CNVs was 53.79 kb and 17.34 kb for gains and losses, respectively. For our control population, we included 3,074 Western European patients producing a total of 161,910 CNVs, which had a median CNV count of 18 gains and 34 losses per sample. Although the median and the standard deviation (SD) of the CNV number per sample were similar between the control and case, one brain cancer patient showed a very high CNV count (701 CNVs), resulting in a very large standard deviation (SD = 40.7) for the GBM category. The standard deviation for GBMs becomes comparable (SD = 20.7) to those of other tumor types and controls when it is recalculated without this patient. Summaries of the dataset and characteristics are described in Table 1.

**Table 1:** **Characterization of CNVs for each cancer and control sets**

**Tumor type**	**Samples**	^**a**^ **Filtered samples (blood/adjacent)**	**CNVs**	^**b**^ **CNVR (no)**	**CNV size (bp, mean)**	^**c**^ **Median**	**Mean**	**SD**
Breast (BRCA)	555	336 (270/66)	15836	9440	105943	46	47.1	14.2
Brain (GBM)	524	351 (344/7)	18072	9286	111178	45	51.5	40.7
Colon (COAD)	444	342 (262/80)	17830	9463	93777	49	52.1	17.1
Kidney (KIRC)	505	370 (51/319)	17968	9574	99424	47	48.6	13.7
Ovarian (OV)	514	380 (314/66)	19204	9777	95492	47	50.5	26.1
Control (MIGEN)	3074	2956	161910		79389	52	54.8	20.5

^a^Filtered samples are the number of cases after the exclusion of low-quality samples. The cases are categorized according to their source of normal tissues (blood or adjacent normal tissues). ^b^CNVR are the number of CNVRs assessed in each of the five association studies with control (MIGEN). ^c^The median, mean and standard deviation (SD) of the number of CNVs per individual are shown.

To merge individual CNVs into common CNV regions (CNVR), we used CNVruler [28], which is one of the few tools that integrate multiple methods for calculating CNVRs, with several statistical association tests and options for population stratification. To identify regions significantly associated with risk of individual cancers, the frequencies of specific CNVRs were compared between each cancer cohort and the MIGEN control group using Fisher’s exact test. To detect potential association, we used the significance level of *P* < 1 × 10^−4^. Given that the number of CNVRs is generally smaller than 500, this p-value threshold is more conservative than the Bonferroni correction on *P =* 0.05. Amplified and deleted CNVRs were calculated separately. A total of 75 rare and common CNVRs were significantly associated with at least one of the cancers studied.

To assess their possible phenotypic impact, we compared the CNVRs to known genomic imbalances collected in the Database of Genomic Variants (DGV) [29]. The DGV release we utilized includes 290,000 CNVs from 8802 samples compiled from 53 studies, covering 66.5% of the human genome [4]. In principle, cancer-specific CNVRs found in DGV reduces the likelihood of the region being causative. However, since DGV is not a curated database and integrates data from multiple platforms with significantly varying probe coverage and resolutions, many variants are known to have inaccurate boundaries, overestimated sizes [24,30] and misleading frequencies [31], while regions identified in many studies or by multiple independent methods are most likely real. We therefore filtered common CNVRs found in multiple studies and samples from DGV and obtained a total of 39 rare CNVRs that are associated with cancer risk for the five cancers. The full list of significant germline CNVRs is shown in Table 2.

**Table 2:** **Significant cancer germline copy number variable regions**

**Cancer**	**Chr**	**Start**	**Size**	**Freq (control)**	**Freq (case)**	**Type**	**OR**	**P-value** ^**a**^	**P-value (PCA)** ^**b**^	**Gene(s)** ^**c**^
BRCA	11	51185363	8472	0%	1.49%	Loss	-	1.08E-05	9.66E-01	
BRCA	3	62936471	30079	0.07%	1.79%	Loss	26.85	2.54E-05	1.05E-04	
BRCA	3	26586501	3489	0.17%	2.08%	Loss	12.56	5.47E-05	3.44E-06	
COAD	3	107601890	16832	0.03%	2.34%	Loss	70.78	1.02E-07	4.90E-05	
COAD	10	101261779	22068	0%	1.46%	Loss	-	1.20E-06	9.63E-01	*NKX2-3*
COAD	4	156797864	71044	0.24%	2.34%	Loss	10.09	4.13E-05	9.20E-06	*GUCY1A3*
COAD	7	29635116	120414	0.03%	1.46%	Gain	43.84	6.41E-05	6.13E-04	*DPY19L2P3, LOC100271874, LOC646762*
GBM	14	21685305	117313	0.30%	5.41%	Loss	18.74	5.54E-13	6.54E-12	*TRA@, TRD*
GBM	5	57361784	7507	16.50%	33.00%	Loss	2.5	1.54E-12	2.52E-20	
GBM	22	47288391	152640	0.07%	2.85%	Loss	43.31	8.76E-09	6.97E-06	*FAM19A5*
GBM	7	38257218	88038	0.84%	5.41%	Loss	6.71	2.25E-08	1.24E-09	*TARP*
GBM	5	10927644	15240	0%	1.99%	Loss	-	1.44E-07	9.60E-01	
GBM	14	21804698	2132	0.10%	2.28%	Loss	22.96	1.85E-06	1.42E-05	*TRA@, TRD*
GBM	14	21681152	2379	0.20%	2.56%	Loss	12.94	4.34E-06	6.42E-06	*TRA@, TRD*
GBM	10	42882051	56351	0.30%	2.56%	Loss	8.62	3.15E-05	3.75E-06	*RET*
GBM	7	61793773	26492	1.56%	5.13%	Loss	3.42	6.89E-05	5.00E-07	
KIRC	14	21681152	2379	0.20%	5.41%	Loss	28.1	6.44E-15	3.49E-12	*TRA@, TRD*
KIRC	10	96855083	4614	0.07%	3.24%	Loss	49.51	2.26E-10	1.23E-07	
KIRC	3	89250592	142689	0%	1.62%	Gain	-	1.83E-06	9.63E-01	*EPHA3*
KIRC	2	97429511	99111	2.17%	7.03%	Loss	3.42	2.33E-06	1.26E-08	*ANKRD36B*
KIRC	6	118470482	5095	0.24%	2.43%	Loss	10.5	1.33E-05	3.52E-06	*SLC35F1*
KIRC	17	34990311	173216	0.64%	3.51%	Gain	5.63	1.56E-05	1.54E-05	*C17orf37, ERBB2, GRB7, NEUROD2, PGAP3, PNMT, PPP1R1B, STARD3, TCAP*
KIRC	4	103363913	68353	0.10%	1.89%	Loss	18.99	1.78E-05	4.83E-05	*SLC39A8*
KIRC	2	91049141	1293	0.58%	3.24%	Loss	5.79	2.68E-05	3.67E-06	
KIRC	4	2281	109282	3.45%	8.38%	Gain	2.56	5.16E-05	2.94E-06	*ZNF595, ZNF718*
KIRC	7	19542080	79082	0.04%	1.35%	Loss	40.48	9.06E-05	6.47E-04	
KIRC	12	130123182	31743	0.04%	1.35%	Loss	40.48	9.06E-05	1.11E-03	*GPR133*
OV	13	54589383	6308	0.07%	2.37%	Loss	35.83	1.32E-07	1.13E-05	
OV	4	36584413	19612	0.03%	2.11%	Loss	63.55	2.15E-07	1.52E-04	
OV	1	244904225	32016	0%	1.84%	Gain	-	2.37E-07	9.60E-01	
OV	10	66977929	15004	4.57%	11.60%	Gain	2.74	3.29E-07	3.64E-11	
OV	2	192993	16566	0%	1.58%	Gain	-	2.11E-06	9.63E-01	*SH3YL1*
OV	1	229982231	47730	0%	1.58%	Gain	-	2.11E-06	9.63E-01	*DISC1, DISC2, TSNAX-DISC1*
OV	2	7529134	41988	0%	1.58%	Gain	-	2.11E-06	9.63E-01	
OV	10	495985	75956	0%	1.32%	Gain	-	1.87E-05	9.47E-01	*DIP2C*
OV	5	174076632	49822	0%	1.32%	Gain	-	1.87E-05	9.47E-01	*MSX2*
OV	18	48381779	37120	0%	1.32%	Gain	-	1.87E-05	9.48E-01	*DCC*
OV	18	45329306	46009	0%	1.32%	Gain	-	1.87E-05	9.47E-01	*LIPG*
OV	4	172611459	3050	4.63%	10.00%	Loss	2.29	6.75E-05	1.95E-09	

^a^The P-value is based on two-tailed Fisher’s exact test comparing gain and loss frequency in cases versus controls using a threshold of 10^−4^. ^b^The significance estimated in a regression analysis using the first component of principal component analysis as covariates. ^c^Overlapping genes with CNVRs (in either case or control) were determined using Refseq as the annotation source.

There are over 200 inherited cancer syndromes that account for 5-10% of all cancer cases [32]. However, all known cancer susceptibility genes account for only 1% to 15% of familial cancers [33]. Therefore, a large fraction of variants that increase genetic predisposition in hereditary cancers remains to be uncovered. Common CNVRs are unlikely to be associated with disease [34], but highly penetrant rare CNVRs are likely to increase cancer susceptibility [30]. With our larger sample size compared to previous case–control association studies, we have greater statistical power to identify novel germline CNVRs associated with cancer.

### Breast invasive carcinoma

Breast cancer is the most common female malignancy in the world, with more than 1.3 million cases and over 450,000 deaths each year [35]. One in eight women in the United States is diagnosed with breast cancer and it accounts for 30% of all female cancers [36]. It is a complex genetic disease where up to a quarter of all cases are likely to be hereditary [33]. Genomic gains and losses in *BRCA1/BRCA2* have been reported to increase predisposition for hereditary breast and ovarian cancers [11,12,37]. CNVs at 17q11.2, 11q13.1, and 6q24.1 were recently reported to be strongly associated with breast cancer recurrence [38]. Inheritable syndromes including Li-Fraumeni syndrome (LFS) and Peutz-Jeghers syndrome (PJS) have genomic rearrangements in *TP53* and *STK11*, respectively, that increase risk of early onset cancers including breast [39,40]. Clinically relevant mutations in *BRCA1*, *BRCA2*, *TP53*, and *PTEN* are well recognized but only account for 5-10% of all new cases, leaving a large fraction of genetic predisposition to be uncovered [41].

Our analysis of germline CNVs for 336 breast cancer patients revealed 10,408 CNVs as losses and 5,428 as gains (median count of 15 gains and 31 losses per individual). We found three CNVR losses significantly enriched in the germline of breast cancer patients: 11p11.12, 3p14.2, and 3p24.1. The deletion at 11p11.12 was detected in five breast cancer patients (the length of CNVR is 8 kb) but not observed in the control set (*P* = 1.08 x 10^−5^). The deletion at 3p14.2 (30 kb in length) was observed in 1.8% (6/336) of cases and 0.07% (2/2956) in the control population (*P* = 2.54 x 10^−5^, odds ratio (OR) = 26.85). The deletion at 3p24.1 was 3 kb in length and was observed in 2.1% (7/336) of the cases and 0.16% (5/2956) in the controls (*P* = 5.47 x 10^−5^, OR = 12.57). None of these deletions showed overlap with known coding sequences. In addition, we analyzed the association between the observed germline variants and disease subtypes (luminal A and B, basal, and HER2 molecular subtypes as reported by the TCGA consortium) [15]. Among the three susceptible loci, the deletion at 3p24.1 were observed only for the patients categorized as luminal A type (*P* = 0.0339, Fisher’s exact test).

### Colorectal cancers

Colon cancer is the fourth most commonly diagnosed malignancy and the second leading cause of cancer-related mortality worldwide with a 6% lifetime risk in the United States [36]. The present estimate is that 15–30% of cases may have a major hereditary component [42,43]. CNVRs associated with colon cancer have been found in multiple inherited colorectal tumor syndromes: large deletions in *APC* confer increased risk for patients with familial adenomatous polyposis coli (FAP) [44]; a CNVR at 3p26 is associated with *APC* mutation negative familial colorectal cancer [10]; hereditary non-polyposis colorectal cancer (HNPCC or Lynch syndrome) accounts for 5% of colon cancers with predisposing CNV deletions in *PMS2*, *MLH1*, *MSH2,* and *MSH6* [37,45-48]; and genomic rearrangements in *STK11* increase risk of early onset cancers including colon in patients with Peutz-Jeghers syndrome (PJS) [40].

**Figure 2:**
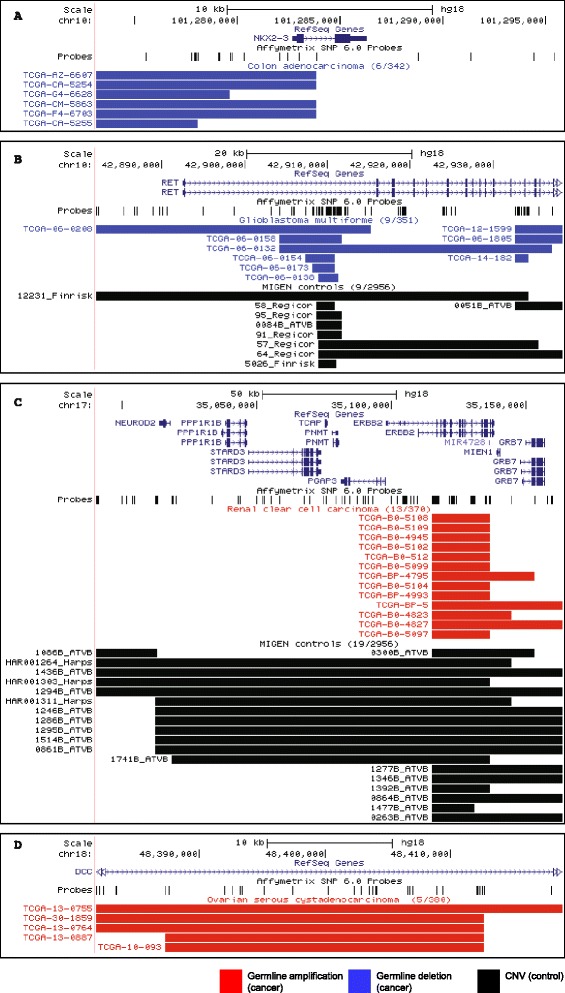
**Illustrative examples of rare germline CNVRs over-represented in specific cancers.** In each panel, the first two tracks after the genomic coordinates show the RefSeq gene annotations and the positions of the probles on the Affymetrix SNP6.0 arrays. Below that, germline CNVs for cancers cases are indicated in blue (losses) and red (gains), followed by CNVs observed in control individuals in black. **(A)** 22 kb loss affecting *NKX2-3* in 6 colorectal cancer cases (n = 342); none is present in the controls (n = 2956). **(B)** 56 kb loss affecting *RET* in 9 glioblastoma patients; 9 CNVs are also found in the controls but the sample size of the control set is almost 10-fold greater (351 vs 2956), making this statistically significant. **(C)** 173 kb gain affecting *ERBB2* in 13 kidney cancer cases (n = 370); 19 are present in the controls. **(D)** 37 kb gain affecting *DCC* in five ovarian cancer cases (n = 380); none are present in the controls.

We identified 12,031 CNVs as losses and 5,799 as gains from 342 genomes of colon cancer patients (median count of 15 gains and 34 losses per individual). Four significant regions associated with colon cancer were identified at 3q13.11, 10q24.2, and 4q32.1 as losses and at 7p15.1 as a gain. The most significant deletion of 3q13.11 was 16 kb in length and did not overlap with any coding sequences. It was observed in 2.3% (8/342) of cases and 0.03% (1/2956) of controls (*P* = 1.02 x 10^−7^, OR = 70.78). The 10q24.2 deletion was 22 kb in size and occurred in 1.75% (6/342) of cases but not observed in the control set (*P* = 1.2 x 10^−7^). Of the six cases, a loss involving the first exon and 5′ untranslated regions (UTR) of *NKX2-3* was observed for four patients (Figure 2A). *NKX2-3* encodes a homeodomain containing a transcription factor. Its variants have been previously reported to be associated with inflammatory bowel diseases, the premalignant disorder of colorectal cancers [49,50]. The deletion at 4q32.1 involving *GUCY1A3* was observed in eight colorectal cancer patients (*P* = 4.13 x 10^−5^, OR = 10.09). Large CNVs (~120 kb) on gain of 7p15.1 encompassing several genes *DPY19L2P3*, *LOC100271874*, *LOC646762* were observed in five colorectal patients (1.46%) while only observed in the control set once (*P* = 6.41 x 10^−5^, OR = 43.84).

### Glioblastoma multiforme

Glioblastoma multiforme (GBM) is the most deadly subtype of brain tumors in adults. In 2012, 22,910 Americans were estimated to have been diagnosed and 13,700 were estimated to have died from brain and other nervous system cancers [36]. GBMs are currently incurable and are responsible for a disproportionately share of cancer mortality with patients typically surviving less than 18 months [51]. Approximately 5% of patients have a family history including rare genetic syndromes including Li-Fraumeni syndrome where genomic rearrangements in *TP53* were associated with elevated brain cancer risk [39]. Germline duplications in *SMARCB1* are associated with increased risk of malignant rhabdoid tumors (MRT) found in the brain [52]. It has also been suggested that hemizygous germline deletions of 22q are possible predisposition loci for GBM [53].

We identified 12,875 CNVs as losses and 5,197 as gains (median count of 13 gains and 32 losses per individual) from 351 genomes of GBM patients. A total of nine significant CNVRs associated with brain malignancies were observed. All associations were identified as genomic losses. Four deletions (one at 7p14.1 and three at 14q11.2) overlapped with genomic loci encoding T cell receptors (*TCR*) including the most significant involving *TCR-alpha* that occurred in 5.4% (19/351) of cases 0.3% (9/2956) of controls (*P* = 5.54 x 10^−12^, OR = 18.74). These deletions overlap with known regions associated with less aggressive forms of neuroblastoma [14]. The deletions at 5q11.2 were recurrent in case (33%; 116/351) and control populations (16.5%; 487/2956) (*P* = 1.54 x 10^−12^, OR = 2.5), but did not involve known coding regions. The deletion observed at 22q13.32 overlaps with *FAM19A5*, the association of which was previously observed with pancreatic cancers [54]. This deletion was observed in 2.8% (10/351) of cases and 0.07% (2/2956) of controls (*P* = 8.76 x 10^−9^, OR = 43.31). Other deletions associated with GBM (~15 kb at 5p14.2 and ~26 kb at 7q11.21) did not involve coding regions. The association of deletions involving the *RET* proto-oncogene with GBM was observed. The deletions occurred in 2.6% (9/351) of cases and 0.3% (9/2956) of the controls (*P* = 3.15 x 10^−5^, OR = 8.62) (Figure 2B). *RET* encodes a receptor tyrosine kinase, which plays an important role in neural development [55] and has been implicated in neuroblastoma [56] and thyroid cancers [57]. Given the presumptive oncogenic role of RET in those tumors, how the germline *deletion* of *RET* may serve as a susceptibility locus is not clear. It is possible that the germline dosage changes of *RET* may have broad implications as shown for their association with Hirschsprung’s disease [58] or this variant is linked to other causal genomic loci. It has also been shown in several examples that the same gene may act as an oncogene or a tumor suppressor depending on its cellular context [59-62].

### Renal clear cell carcinoma

Renal clear cell carcinoma is the most common type of kidney cancer, which accounts for 3-5% of all adult malignancies [36]. It is the sixth most common in cancer in men and eighth most common in woman. Approximately 2-3% of cases are hereditary, including several autosomal dominant syndromes [63]. Germline deletions in *VHL* are associated with Von Hippel-Lindau (VHL) disease, which is characterized by the development of multiple vascular tumors including the kidney [64]. Rare full gene deletions of *FH* predispose individuals to hereditary leiomyomatosis and renal cell cancer (HLRCC) [65]. Children with malignant rhabhoid tumors (MRT), a particularly aggressive pediatric kidney cancer, have found germline duplications in *SMARCB1* associated with increased cancer risk [52]. Large genomic deletions and rearrangements in *TSC1* and *TSC2* in tuberous sclerosis contribute to harmartomas found in multiple organs including the kidney [66].

We identified 12,242 CNVs as losses and 5,726 as gains (median count of 15 gains and 32 losses per individual) from 370 genomes of renal cell carcinoma (clear type). Eleven significant CNVRs were associated with kidney cancer, with eight loss CNVRs and three gain CNVRs. The most significant CNVR occurred as a deletion at 14q11.2 involving genomic loci encoding *TCR-alpha* (*P* = 6.44 x 10^−15^, OR = 28.09). The second significant locus was observed at 10q23.33 (*P* = 2.26 x 10^−10^, OR = 49.50) without involving coding sequences. Some of the significant loss CNVRs did involve coding sequences. For example, deletions involving *ANKRD36B* on 2q11.2 occurred in 7.0% (26/370) of cases and 2.16% (64/2956) of controls (*P* = 2.33 x 10^−6^, OR = 3.41). Deletions involving solute carrier family-coding regions were observed at two genomic loci: 6q22.2 (*SLC35F1; P* = 1.33 x 10^−5^, OR = 10.5) and 4q24 (*SLC39A8*; *P* = 1.78 x 10^−5^, OR = 18.98). Five patients showed deletions involving the locus encoding G protein-coupled receptor 133 (*GPR133*), while only one control individual showed it (*P* = 9.06 x 10^−5^, OR = 40.48). The remaining CNVRs observed at 2p11.1 and 7p15.3 (observed in 3.2% and 1.4% of cases, respectively) did not overlap with any genes.

The gains at 3p11.2, 17q12, and 4p16.3 were significantly enriched in kidney cancer patients. A CNVR in 3p11.2 encompassing *EPHA3* was observed for six cancer patients (1.6%) and was not found in the control population (*P* = 1.83 x 10^−6^). A gain at 17q12 overlaps with the cancer-related gene *ERBB2.* Interestingly, germline amplifications in cancer patients are localized to *ERBB2*, while many controls have larger CNVs in the same region (Figure 2C). The biological implication of germline amplification involving *ERBB2* is not well understood. However, the known roles of somatic amplification in certain tumor types such as breast cancer raises a hypothesis that different germline copy numbers of *ERBB2* may be a predisposing factor in the affected individuals. The other gain of 109 kb in 4p16.3 overlapping with *ZNF595* and *ZNF718* occurred in 8.4% (31/370) of cases and 3.4% (102/2956) of controls (*P* = 5.16 x 10^−5^, OR = 2.56).

### Ovarian serous cystadenocarcinoma

Ovarian cancer is the fourth most frequent cancer in woman worldwide. In the United States, approximately 22,910 women will be newly diagnosed resulting in 15,500 deaths per year [36]. At least 10% of ovarian tumors are hereditary and associated with autosomal dominant syndromes [67]. Rare hereditary syndromes including Peutz-Jeghers syndrome (PJS) and Gorlin syndrome have germline deletions in *STK11* and *Patch* genes, respectively, that increase the risk of early onset ovarian cancer [40,68]. Germline copy number variants in *BRCA1* and *BRCA2* are known to increase risk of hereditary breast/ovarian cancers independent of their *BRCA1/BRCA2* mutation status [37,69].

Our analysis revealed a total of 12,612 CNVs as losses and 6,592 as gains in 380 ovarian cancer patients (median count of 15 gains and 32 losses per individual). A total of 12 genomic loci showed significant association with ovarian cancer, nine of which were gains and three were losses. Six of the 12 genomic loci were observed in coding regions. Two gain CNVRs including a 16 kb segment in genomic loci encoding *SH3YL1* (2p25.3) and a 47 kb region overlapping with *DISC1*, *DISC2*, and *TSNAX-DISC1* (1q42.2) were observed at the same frequency of 1.6% (6/380) but not observed in the controls (*P* = 2.11 x 10^−6^). Genomic loci encoding *DIP2C* (chr10), *MSX2* (chr5)*, DCC* (chr18)*,* and *LIPG* (chr18) also showed similar frequencies in the ovarian cancer patients of 1.3% (5/380) but not in the control (*P* = 1.87 x 10^−5^). Among them, the association with *DCC* and ovarian cancer pathogenesis has been previously reported [70] (Figure 2D).

### Additional analysis and limitations of this study

It is possible that some variants are shared in multiple tumors types but their effect sizes are too small to be detected in a single-tumor analysis. When we carry out the same analysis on the aggregate data, we indeed can identify more loci of potential interest. Of the 17 loci identified this way, 8 were found with single-tumor analysis but 9 were not. Of these 9, 4 overlapped with genes, including *TFG*, *TP53TG3* and *HLA* loci. The list is shown in (Additional file 1: Table S1). Moreover, we focused our analysis above on discovering tumor susceptibility markers by selecting genomic variants with OR > 1. But we could also search for potentially protective loci by applying the criterion OR < 1. This analysis results in a list of 17 genomic variants (Additional file 2: Table S2), which are all non-coding.

One way to examine potential impact of identified CNVs is to determine whether the differential copy numbers between the samples that carried a CNV and those that did not resulted in a significant difference in gene expression. Proper analysis of this question, however, requires expression profiles of matched normal tissues, when TCGA data only contain expression levels (either RNA-seq or arrays) of the tumor tissues due to the difficulties of obtaining adjacent tissues for RNA analysis. When we limit our analysis to expression data from tumor tissues, it is unlikely to be informative. For instance, we explored whether the CNV at 17q12 may influence the gene expression of *ERBB2* in the cancer cells, and found that it was not significantly different between the tumors harboring this genomic variants and those without (*P* = 0.546; *t*-test). This result, however, does not imply that the germline variant was not functional, as many factors downstream would have contributed to the *ERBB2* expression in tumor cells.

Our analysis has generated a list of CNVs that are significantly associated with cancer risk based on a large number of samples. However, there are several caveats in this computational study. First, before these genes can be utilized as clinical markers, they need to be further validated with PCR or other assays. Such experimental validation was not possible in our study due to the fact that consortium projects are not able to provide DNA samples for individual studies. Future studies on independent cohorts will also be necessary before these markers can be utilized. Second, although we have paid a great deal of attention to the bioinformatics aspect (e.g., re-processing case and control datasets from raw data to remove computational artifacts), it is possible that some of the CNVs may be rare variants that happened to be present at lower frequency in the particular control dataset we had. This may be due to chance or to any bias that may have occurred in sample collection, including patient characteristics such as race and age. Our selection of Caucasian patients from sample annotations and principal componenet analysis alleviates bias due to differences in population structure, but it may not have been removed completely. Third, it remains possible that the reported variants are not causal variants but are linked to the true causal variants. Functional *in vitro* or *in vivo* studies on the impact of specific CNVs will be needed for a better understanding of causal relationships. Finally, the list derived in this work is clearly incomplete. Although Affymetrix SNP arrays have been extensively used in the field, they are not able to detect small CNVs; many variants were undoubtedly missed also due to the low frequency of many of these CNVs. Subsequent studies on larger populations using exome or whole-genome sequencing data will be needed for more complete lists.

## Conclusion

This study provides a new catalogue of over-represented germline CNVs that potentially contribute to cancer risk, utilizing a publicly available dataset of a large population of cancer patients across multiple cancer types. As expected, most candidate prognostic CNVs we find have low frequencies despite their statistical significance. Among the most interesting cases are the rare germline CNVs affecting *RET* in GBMs, *ERBB2* in renal cell carcinomas, and *DCC* in ovarian cancers. Although causal relationship should be tested in independent cohorts in the future, these CNVs may explain some of the disease heritability not previously identified. Interestingly, few CNVs associated with disease risk are shared among cancers, suggesting that either there is a diversity of pathways through which germline CNVs confer cancer risks or our sample size is still too small to detect such low frequency events. Further studies profiling other germline characteristics, such as epigenetic alterations and combined effects of multiple variants, will also be helpful for a more comprehensive understanding of cancer predisposition.

## Methods

### Sample selection

Genotyping was performed using the Affymetrix SNP 6.0 arrays in the TCGA consortium. Cases are germline-derived DNA samples (peripheral blood or adjacent tissues) of European ancestry; raw . CEL files were downloaded from The Cancer Genome Atlas Data Portal (https://tcga-data.nci.nih.gov) in May 2012. Control samples were obtained from the Myocardial Infarction Genetics Consortium (MIGEN) (phs000294.v1.p1) [20]. Raw CEL files for healthy controls of European ancestry (n = 3,074) were kindly provided by the investigators of that project.

### CNV detection

CNVs were called using the PennCNV-Affy6 protocol (2011 Jun16 version) on genome build hg18 (http://www.openbioinformatics.org/penncnv/). PennCNV uses a hidden Markov model that incorporates Log R Ratio (LRR) values, B Allele Frequency, SNP spacing, and population frequency to generate CNV calls for each sample [27]. Low quality samples were eliminated from subsequent analysis using defaults in PennCNVs *filter_cnv.pl* program in addition to filtering samples with a standard deviation of normalized intensity (LRR) > 0.35. The LRR is a normalized measure of total signal intensity for two alleles of a SNP.

### CNVR detection and association testing

Illustrative examples of germline CNVRs are shown in Figure 2 with the remaining CNVRs (in Table 2) illustrated in (Additional file 3: Figure S1). CNVRuler (v1.3) was used to merge individual CNVs into common CNV regions (CNVR) for each cancer and control set [28]. CNVRs that did not have a recurrence of > 0.1 were filtered from the list. CNVR frequencies between each cancer set and controls were evaluated using two-tailed Fisher’s exact test. Significant (*P* < 1.0 x 10^−4^) differences were considered as potential associations. The significance of association was also calculated by using the first component from PCA as a covariate in CNVRuler. PCA analysis was performed using the CNV calls on the CNVR markers for each of the five cancer types [71]. The scatter plots of the first and second principle components show no population stratification in the controls and cases for the five cancer types (Additional file 4: Figure S2). The overlap with known CNVs was determined by counting the number of times each CNVR was observed in the Database of Genomic Variants (DGV) using Release 2012-03-29 [4]. Since CNV boundaries defined by DGV are known to be variable and not entirely accurate, we classified CNVRs observed in fewer than 100 individuals as a rare event.

## Additional files

Additional file 1: Table S1. Germline variants identified in the pooled cancer cases. Additional file 2: Table S2. Protective genomic loci with OR < 1. Additional file 3: Figure S1. Graphical representation of all the CNVRs. Additional file 4: Figure S2. The first and second principle components in PCA analysis.

## Abbreviations

CNV: Copy number variation
TCGA: The cancer genome atlas
SNP: Single nucleotide polymorphism
DGV: Database of genomic variants
GWAS: Genome-wide association study
MIGEN: Myocardial infarction genetics consortium
CNVR: Copy number variation region
OR: Odds ratio
UTR: Untranslated regions
GBM: Glioblastoma multiforme
SD: Standard deviation
LRR: Log r ratio
