# Supplementary material for: Identification of rare germline copy number variations over-represented in five human cancer types
Source: Mol Cancer. 2015 Feb 3;14:25. doi: 10.1186/s12943-015-0292-6 (PMC4381456; doi:10.1186/s12943-015-0292-6)
Supplement: Additional file 1: Table S1. — Germline variants identified in the pooled cancer cases. [file 12943_2015_292_MOESM1_ESM.docx]

Table S1. Germline variants identified in the pooled cancer cases

| Chr | Start | Size | Type | ^a^OR (Case/control) | P-value | ^b^Overlapping | Genes |
| --- | --- | --- | --- | --- | --- | --- | --- |
| 14 | 21681152 | 349521 | loss | 8.97 (136/27) | 2.698E-34 | Yes | *HNRNPC, RPGRIP1, SUPT16H, CHD8, RAB2B, TOX4, METTL3, SALL2* |
| 7 | 38257218 | 88038 | loss | 5.23 (76/25) | 8.459E-15 | Yes | *STARD3NL, TARP* |
| 9 | 44195483 | 17755 | loss | 1.97 (186/165) | 1.353E-09 | No |  |
| 12 | 33192673 | 5969 | loss | 1.54 (361/418) | 4.424E-08 | No |  |
| 9 | 68120833 | 5608 | loss | 10.09 (24/4) | 1.984E-07 | No |  |
| 3 | 100427148 | 4964 | loss | 1.45 (441/545) | 2.689E-07 | No | *TFG* |
| 4 | 34455255 | 45866 | loss | 1.43 (460/579) | 5.507E-07 | No |  |
| 10 | 96855083 | 10997 | loss | 15.94 (19/2) | 6.892E-07 | Yes |  |
| 7 | 29640265 | 109974 | gain | 25.12 (15/1) | 4.203E-06 | Yes | *LOC646762, MIR550A3* |
| 4 | 36584413 | 19612 | loss | 23.43 (14/1) | 1.056E-05 | Yes |  |
| 3 | 26552044 | 40226 | loss | 6.15 (22/6) | 1.279E-05 | Yes |  |
| 14 | 18611714 | 871985 | loss | 1.36 (430/559) | 1.839E-05 | No | *OR11H12* |
| 16 | 33149454 | 389878 | loss | 1.4 (326/406) | 2.727E-05 | No | *TP53TG3* |
| 11 | 51185363 | 28459 | loss | NA (10/0) | 5.517E-05 | Yes |  |
| 1 | 244904225 | 37274 | gain | NA (10/0) | 5.517E-05 | Yes |  |
| 6 | 32560779 | 173818 | loss | 1.87 (88/80) | 8.828E-05 | No | *HLA-DQA1, HLA-DQB1, HLA-DQA2, HLA-DQB2* |
| 12 | 8443789 | 32151 | loss | 8.37 (15/3) | 9.075E-05 | No |  |

^a^Case number is the number of total individuals across the five cancer types.

^b^The genomic loci overlapping with those listed in Table 2 are indicated.
