# Supplementary material for: Identification of rare germline copy number variations over-represented in five human cancer types
Source: Mol Cancer. 2015 Feb 3;14:25. doi: 10.1186/s12943-015-0292-6 (PMC4381456; doi:10.1186/s12943-015-0292-6)
Supplement: Additional file 2: Table S2. — Protective genomic loci with OR < 1. [file 12943_2015_292_MOESM2_ESM.docx]

| **Table S2. Protective loci with OR < 1** | | | |  |  |  |  |
| --- | --- | --- | --- | --- | --- | --- | --- |
| Cancer | Chr | Start | Size | Cytoband | Type | Odds Ratio (Case / Control) | P-value |
| BRCA | 5 | 12853889 | 37645 | 5p15.2 | gain | 0 (0/153) | 6.91E-08 |
| BRCA | 12 | 30128764 | 6226 | 12p11.22 | loss | 0.23 (9/313) | 1.90E-07 |
| BRCA | 1 | 112497488 | 10254 | 1p13.2 | gain | 0.26 (10/309) | 1.29E-06 |
| BRCA | 18 | 36514418 | 4970 | 18q12.3 | loss | 0.45 (28/494) | 2.67E-05 |
| BRCA | 5 | 12868780 | 4297 | 5p15.2 | loss | 0.47 (29/493) | 7.30E-05 |
| COAD | 12 | 88972574 | 68070 | 12q21.33 | loss | 0.08 (2/184) | 3.67E-07 |
| COAD | 1 | 112487872 | 19870 | 1p13.2 | gain | 0.36 (14/309) | 6.87E-05 |
| GBM | 5 | 12853889 | 37645 | 5p15.2 | gain | 0 (0/153) | 4.41E-08 |
| GBM | 12 | 89015800 | 406 | 12q21.33 | loss | 0.08 (2/184) | 2.39E-07 |
| GBM | 1 | 112497488 | 10254 | 1p13.2 | gain | 0.19 (8/309) | 2.51E-08 |
| GBM | 18 | 36514418 | 4970 | 18q12.3 | gain | 0.29 (13/343) | 6.51E-07 |
| KIRC | 12 | 89015800 | 406 | 12q21.33 | loss | 0.2 (5/184) | 2.16E-05 |
| KIRC | 18 | 36514418 | 4970 | 18q12.3 | gain | 0.25 (12/343) | 5.20E-08 |
| OV | 12 | 89015800 | 406 | 12q21.33 | loss | 0.16 (4/184) | 2.52E-06 |
| OV | 7 | 54353999 | 13 | 7p11.2 | loss | 0.33 (21/434) | 1.17E-07 |
| OV | 18 | 36514418 | 4970 | 18q12.3 | gain | 0.35 (17/343) | 6.00E-06 |
| OV | 5 | 57361784 | 7507 | 5q11.2 | loss | 0.38 (27/487) | 3.42E-07 |
