# Supplementary figures and images for: Identification of rare germline copy number variations over-represented in five human cancer types
Source: Mol Cancer. 2015 Feb 3;14:25. doi: 10.1186/s12943-015-0292-6 (PMC4381456; doi:10.1186/s12943-015-0292-6)

BRCA

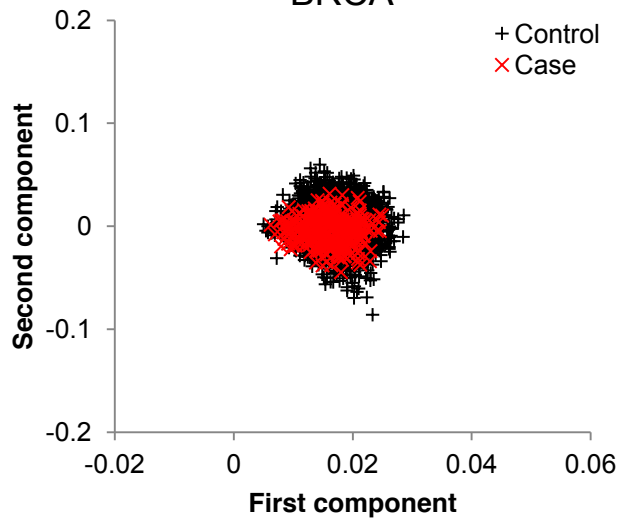

COAD

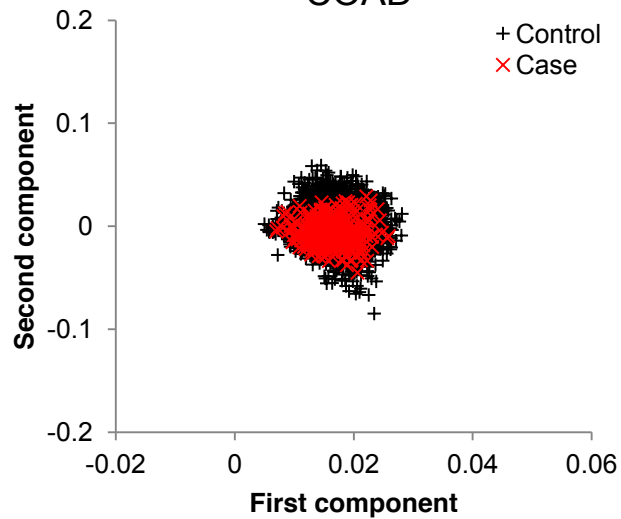

GBM

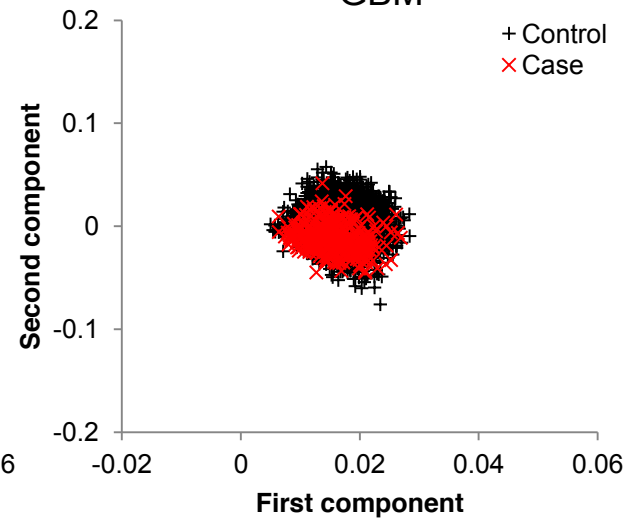

KIRC

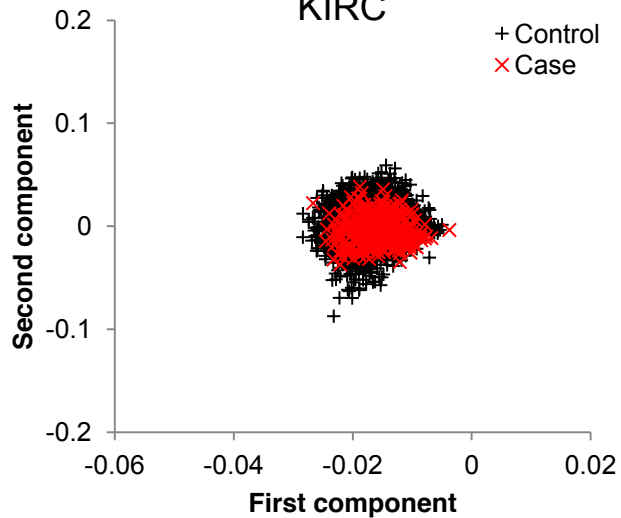

OV

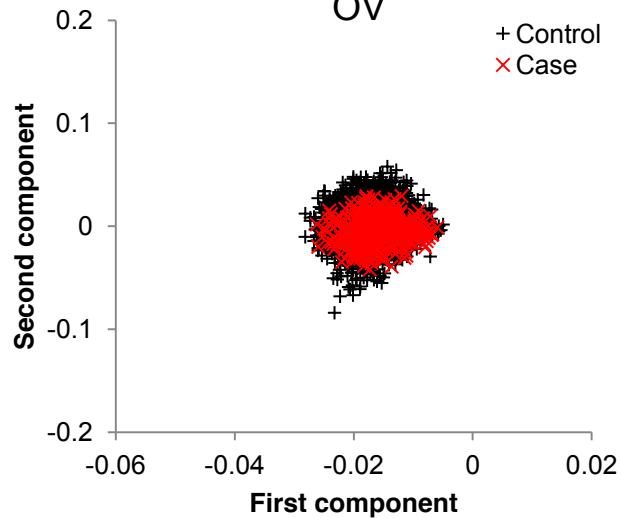

Supplement: Additional file 3: Figure S1. — Graphical representation of all the CNVRs. [file 12943_2015_292_MOESM3_ESM.pdf]
