# Supplementary material for: Identification of rare germline copy number variations over-represented in five human cancer types
Source: Mol Cancer. 2015 Feb 3;14:25. doi: 10.1186/s12943-015-0292-6 (PMC4381456; doi:10.1186/s12943-015-0292-6)

Breast cancers (BRCA), Chr11:51185363

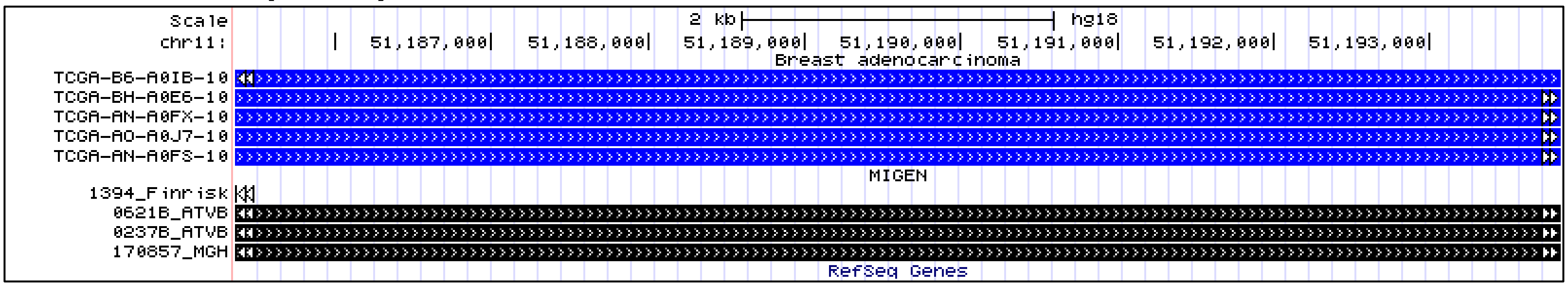

Breast cancers (BRCA), Chr3:62936471

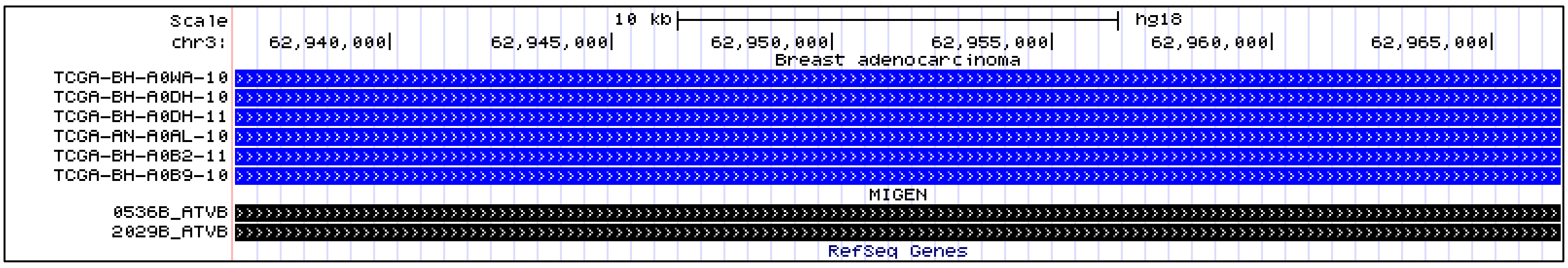

Breast cancers (BRCA), Chr3:26586501

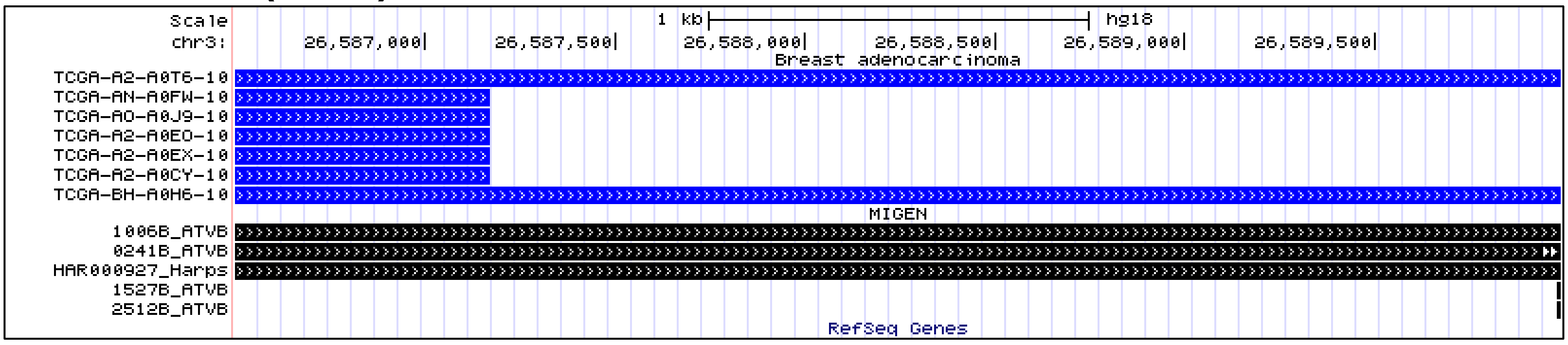

Colorectal carcinoma (COAD, READ), chr3:107601890

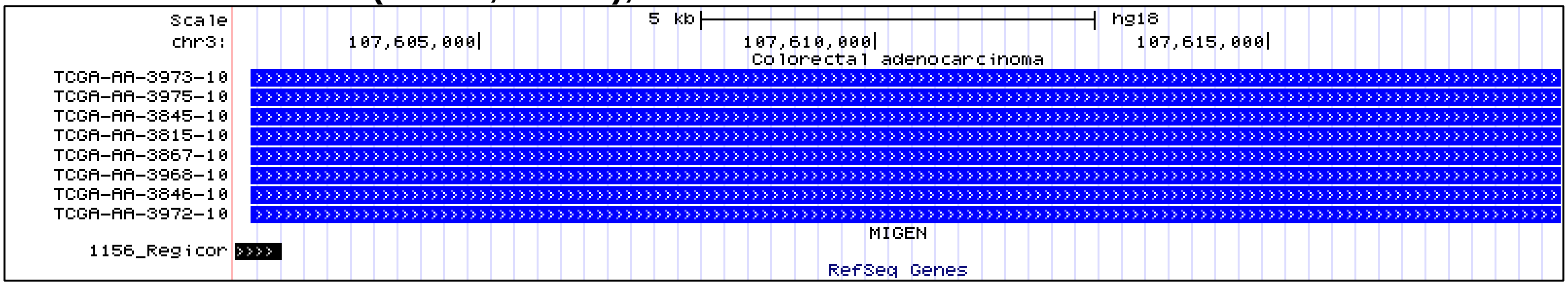

Colorectal carcinoma (COAD, READ), Chr4:156797864

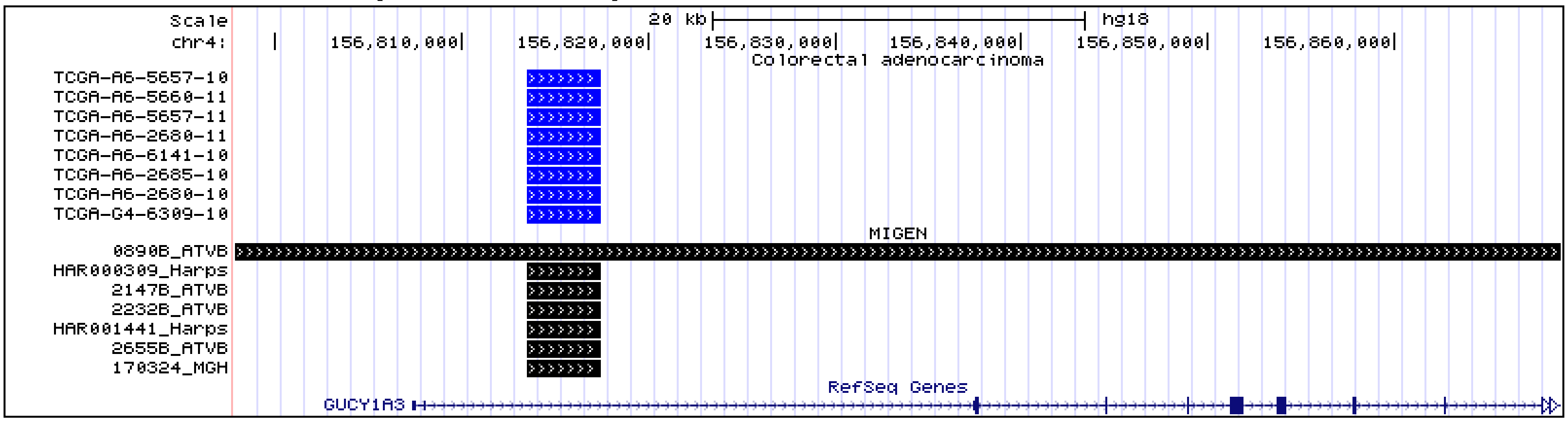

Colorectal carcinoma (COAD, READ), Chr7:29635116

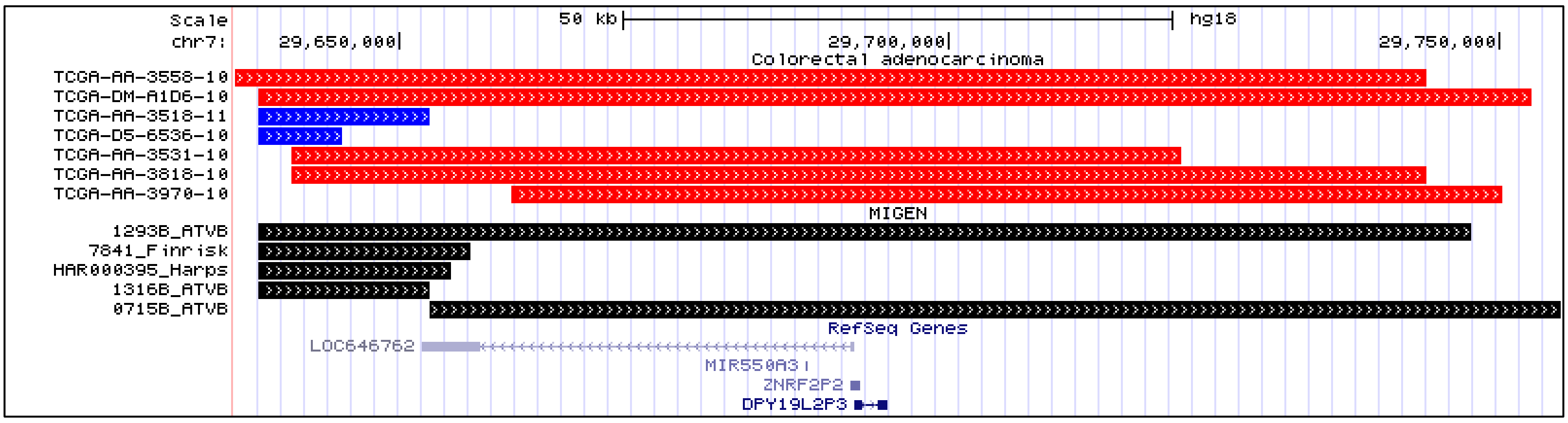

Glioblastoma multiforme (GBM), Chr14:21685305

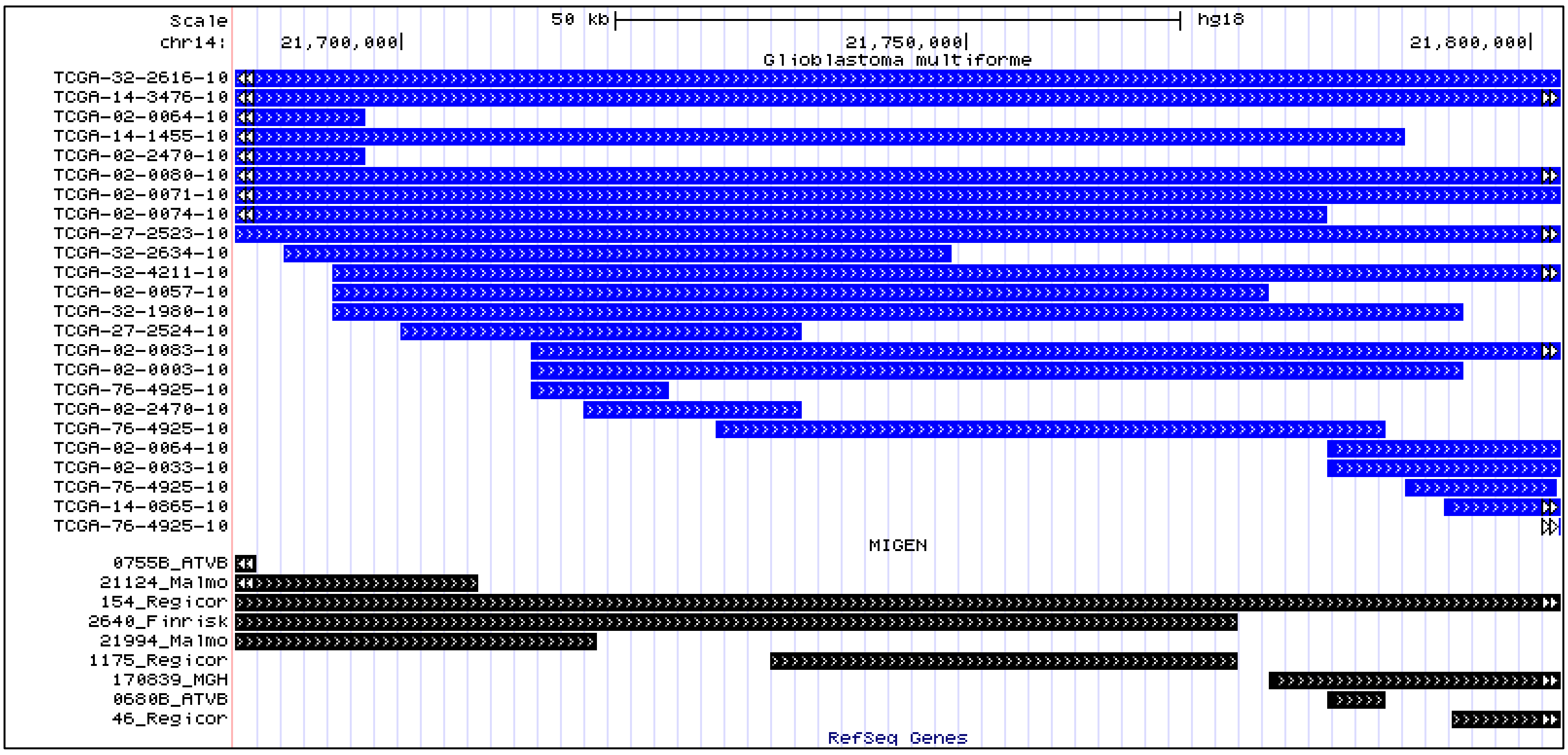

Glioblastoma multiforme (GBM), Chr22:47288391

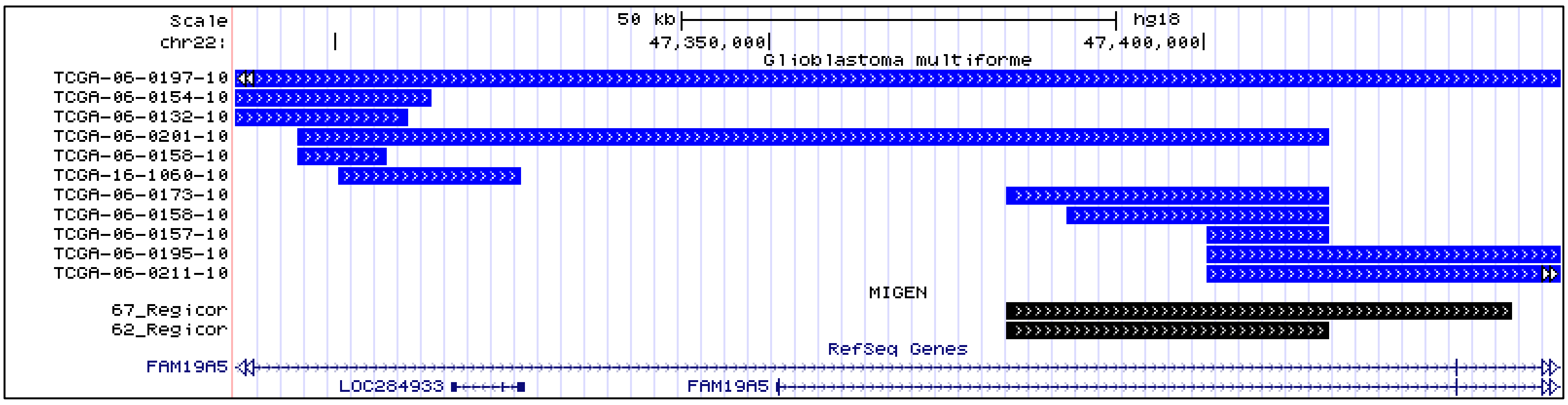

Glioblastoma multiforme (GBM), Chr5:10927644

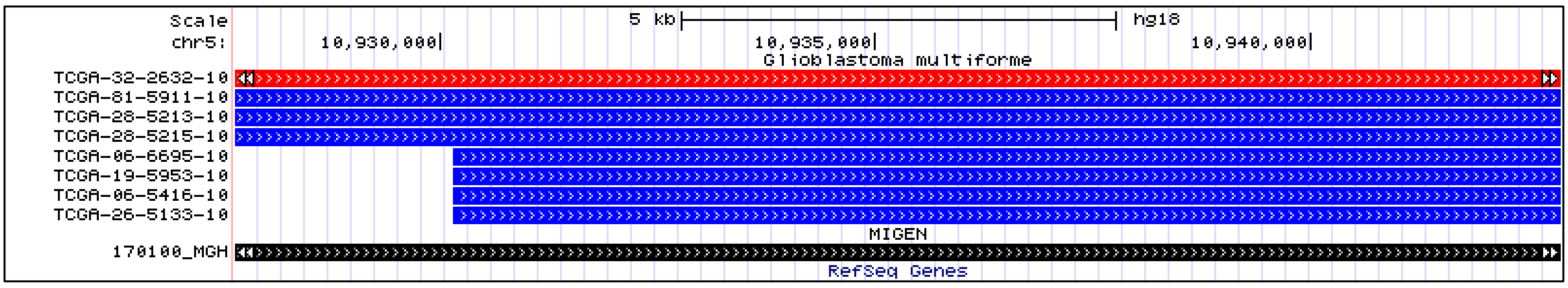

Glioblastoma multiforme (GBM), Chr14:21804698

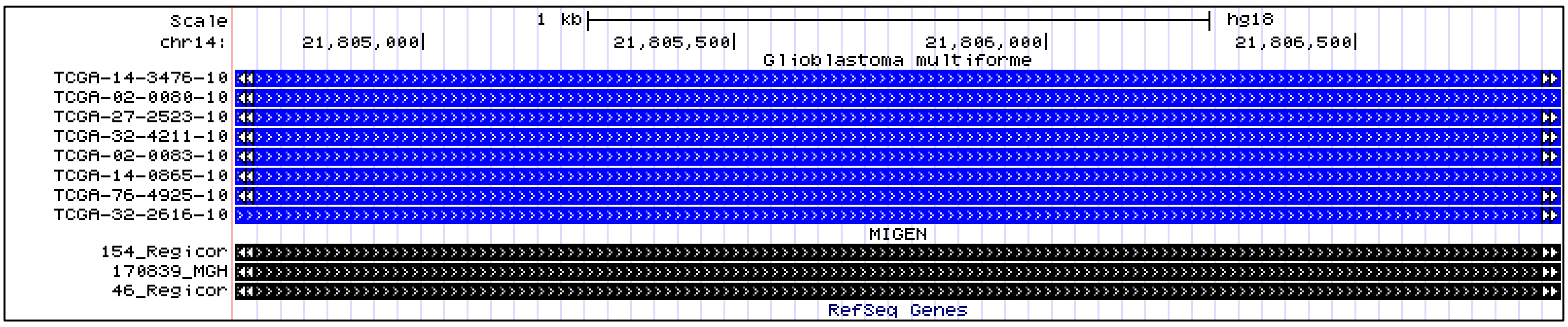

Glioblastoma multiforme (GBM), Chr14:21681152

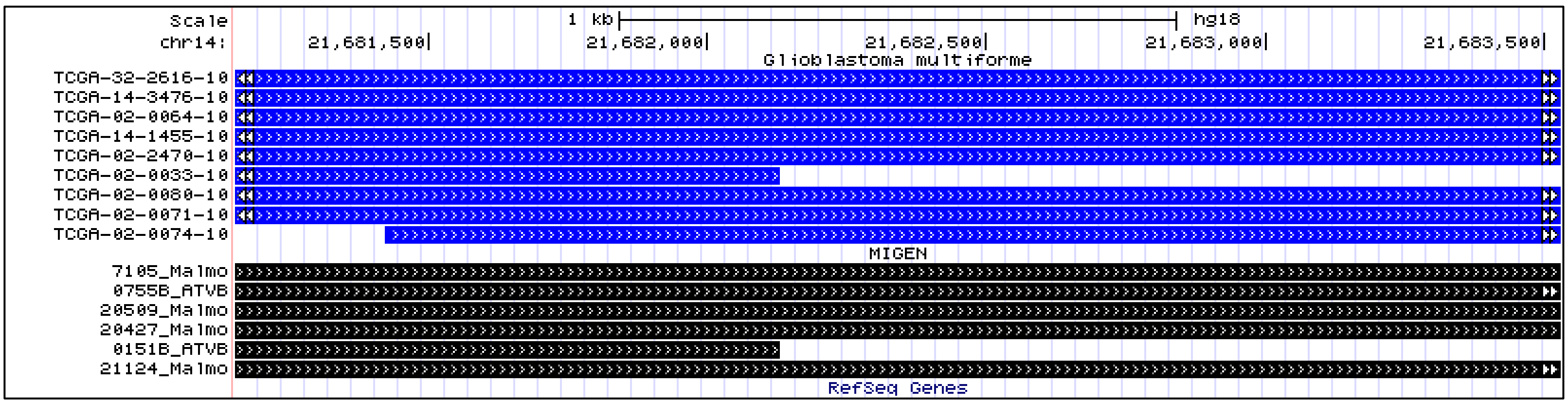

Renal cell carcinoma (KIRK), Chr14:21681152

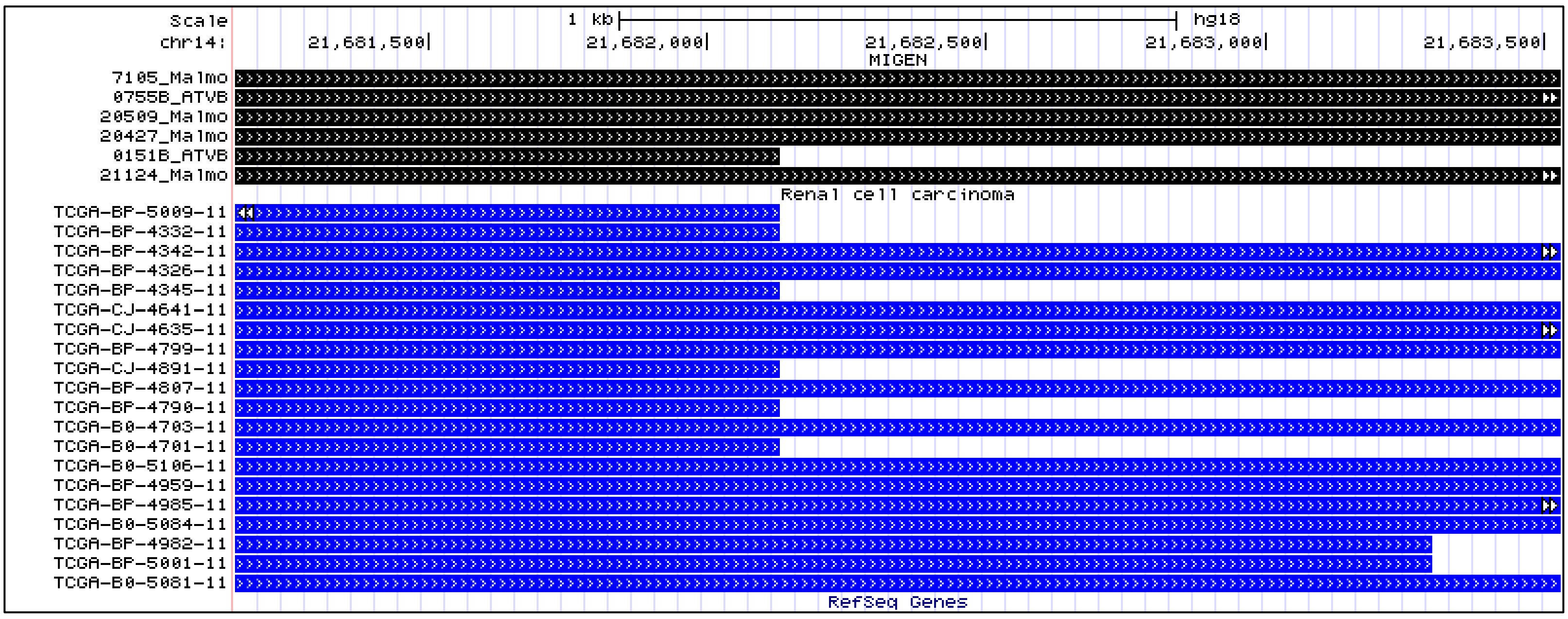

Renal cell carcinoma (KIRK), Chr10:96855083

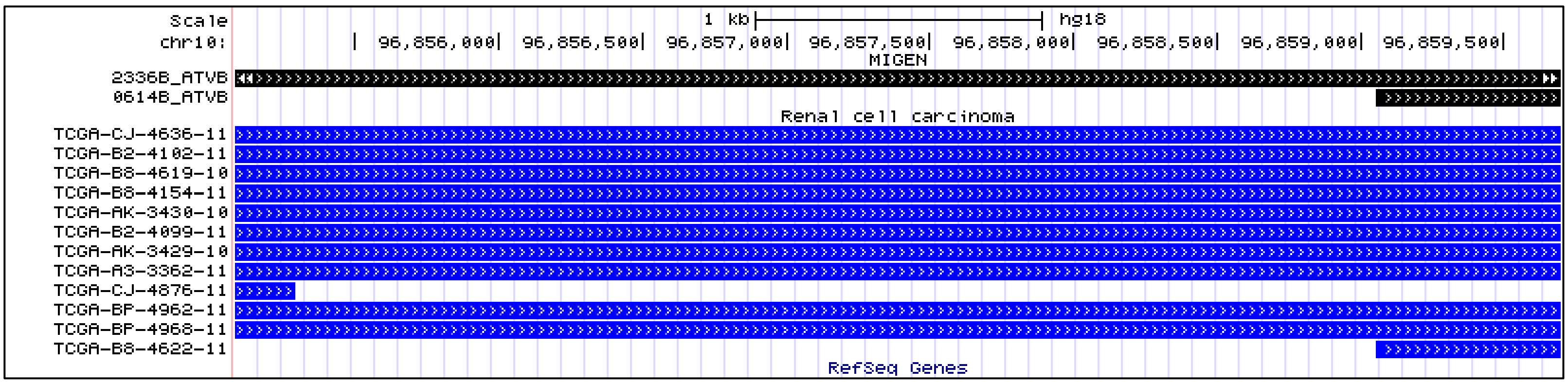

Renal cell carcinoma (KIRK), Chr3:89250592

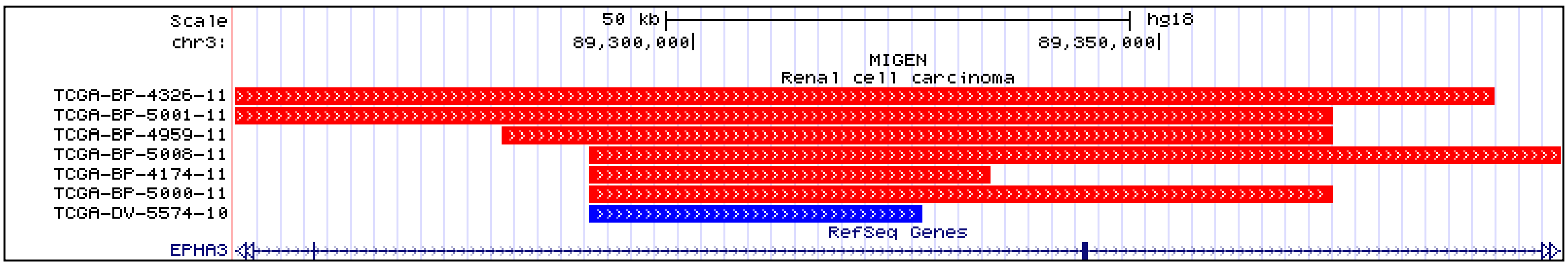

Renal cell carcinoma (KIRK), Chr6:118470482

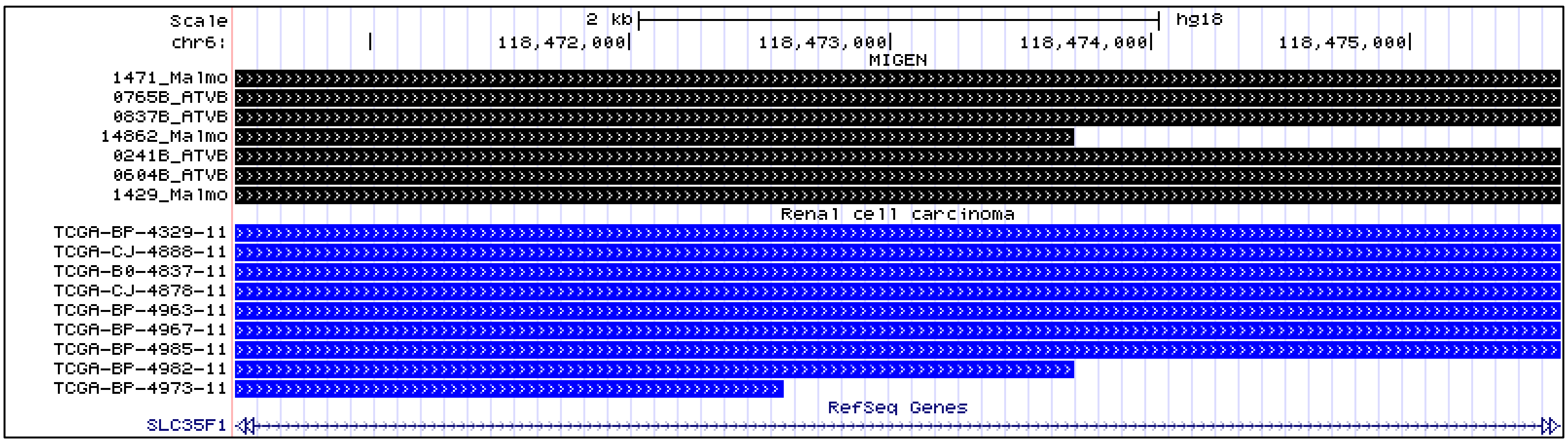

Renal cell carcinoma (KIRK), Chr4:103363913

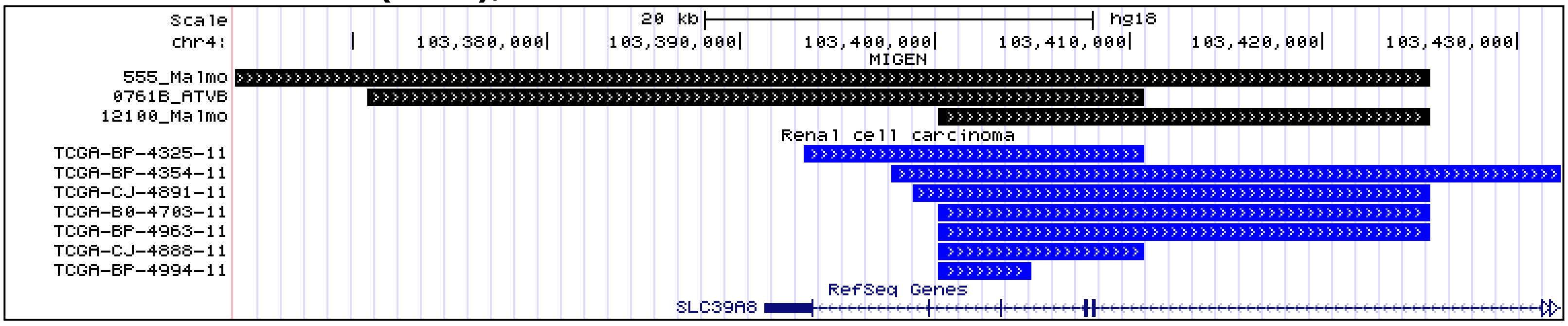

Renal cell carcinoma (KIRK), Chr7:19542080

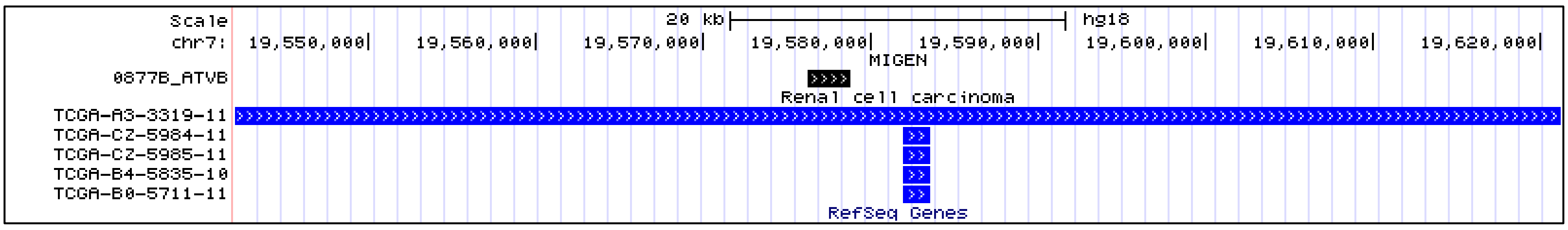

Renal cell carcinoma (KIRK), Chr12:130123182

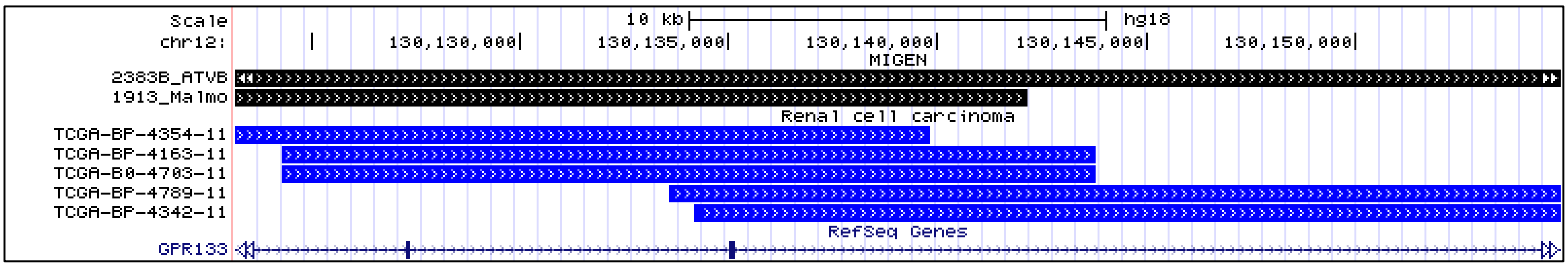

Ovarian cancer (OV), Chr13:54589383

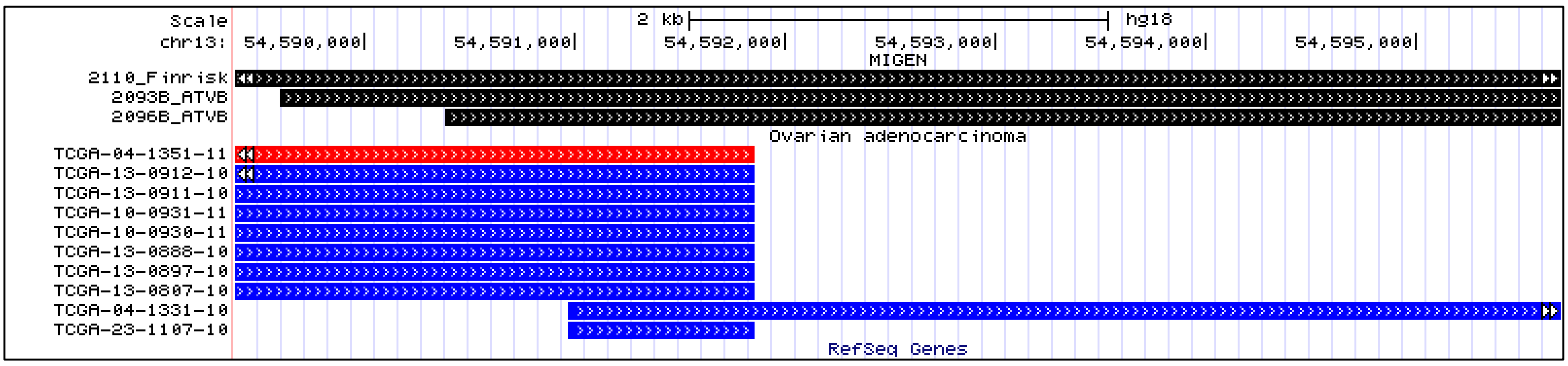

Ovarian cancer (OV), Chr4:36584413

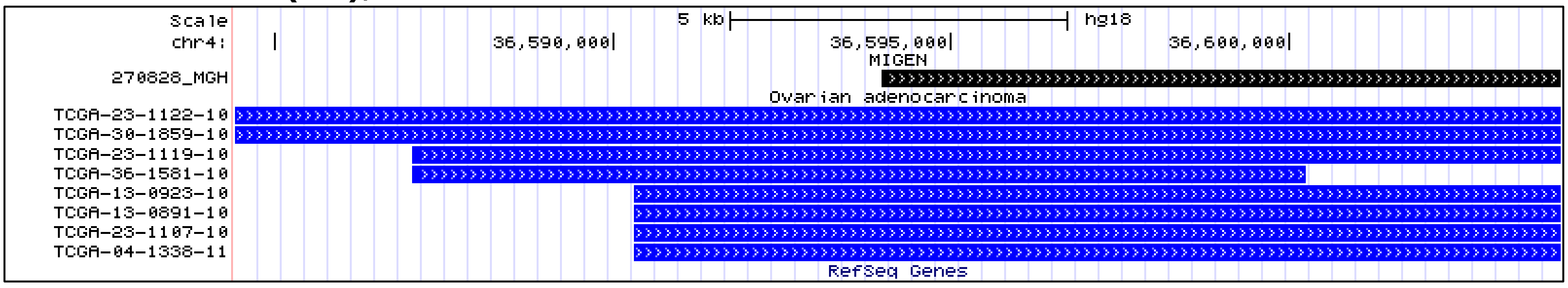

Ovarian cancer (OV), Chr1:244904225

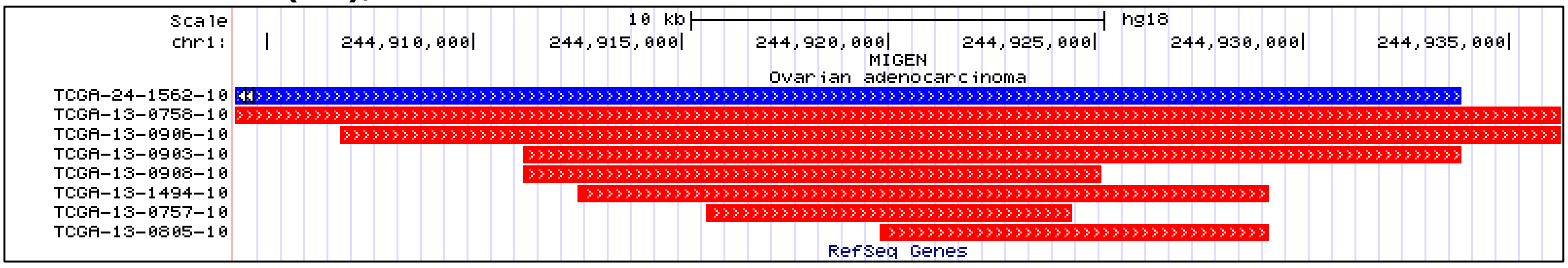

Ovarian cancer (OV), Chr2:192993

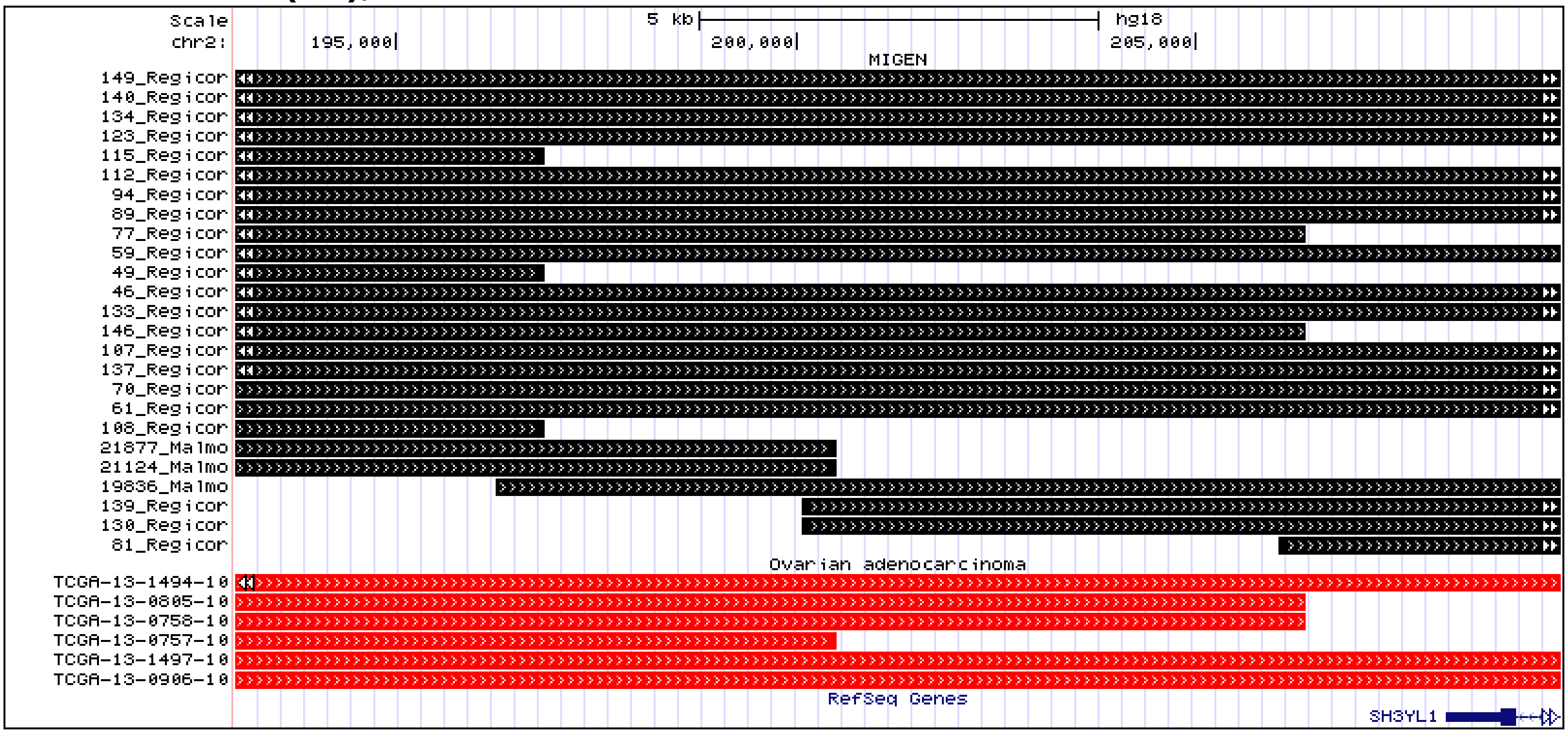

Ovarian cancer (OV), Chr1:229982231

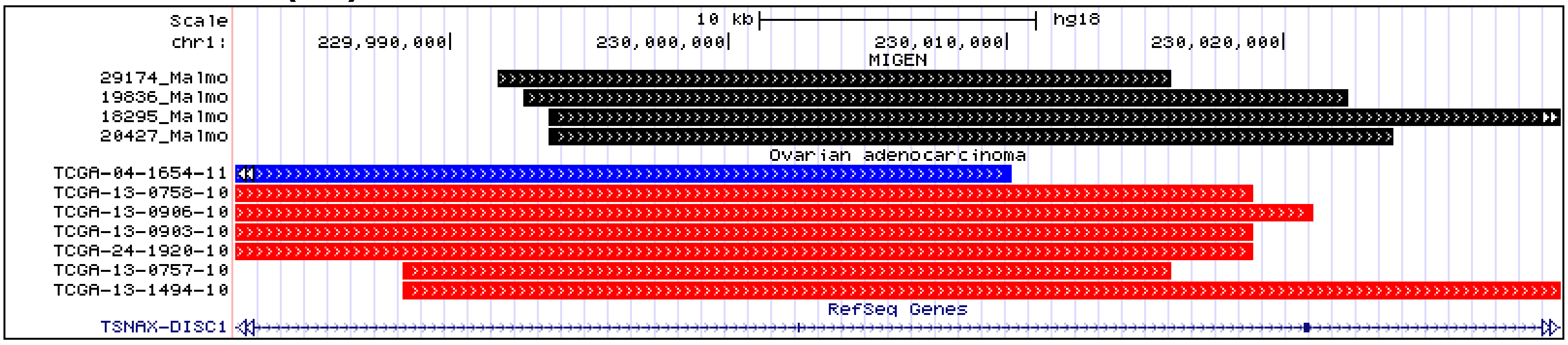

Ovarian cancer (OV), Chr2:7529134

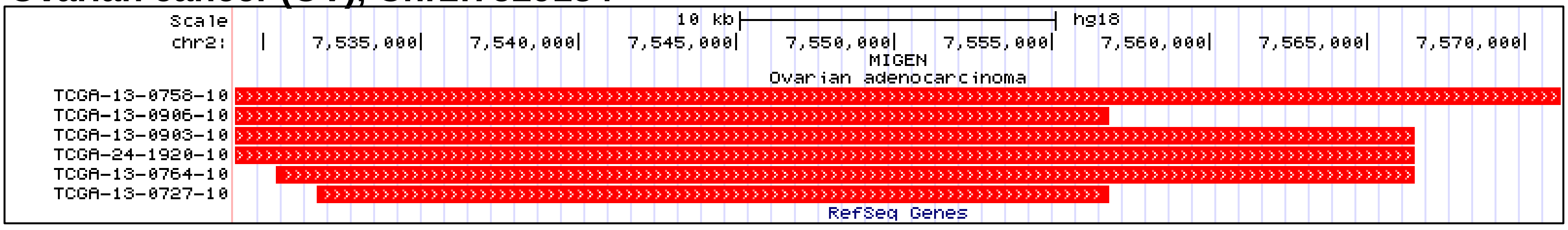

Ovarian cancer (OV), Chr10:495985

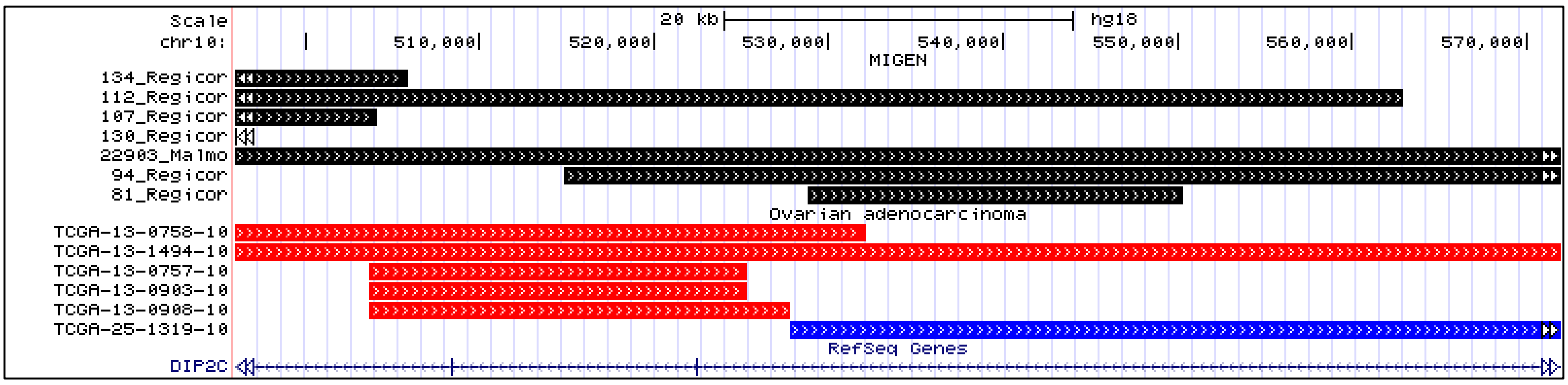

Ovarian cancer (OV), Chr5:174076632

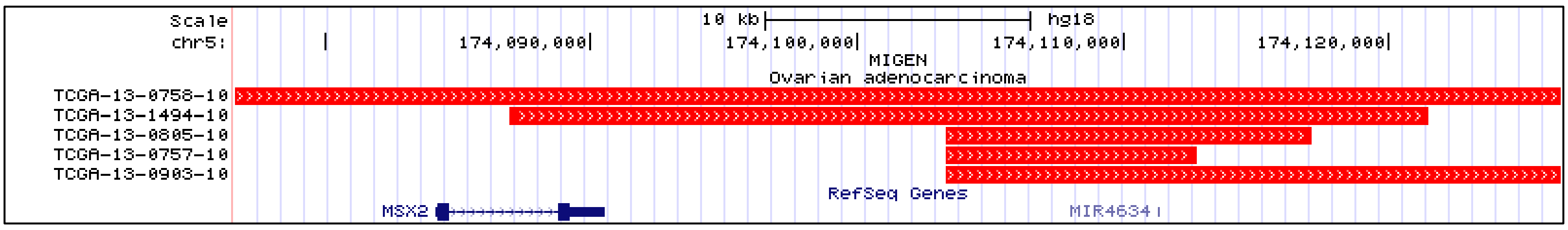

Ovarian cancer (OV), Chr18:45329306

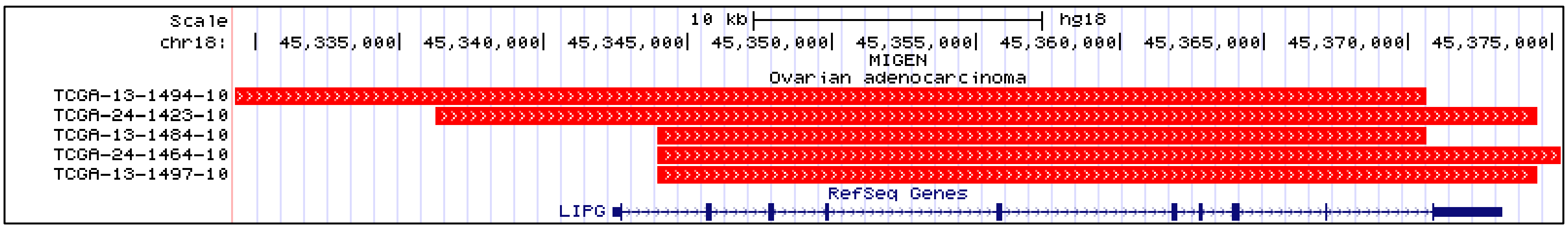

Supplement: Additional file 4: Figure S2. — The first and second principle components in PCA analysis. [file 12943_2015_292_MOESM4_ESM.pdf]
